# Thirteen new species of the genus *Meleonoma* Meyrick, 1914 (Lepidoptera, Autostichidae) from China

**DOI:** 10.3897/zookeys.1270.171167

**Published:** 2026-02-23

**Authors:** Xiaoju Zhu, Shuxia Wang

**Affiliations:** 1 College of Plant Protection, Shandong Agricultural University, Tai’an, Shandong 271018, China College of Life Sciences, Nankai University Tianjin China; 2 College of Life Sciences, Nankai University, Tianjin 300071, China College of Plant Protection, Shandong Agricultural University Tai’an China

**Keywords:** Gelechioidea, Microlepidoptera, species group

## Abstract

Thirteen new species belonging to the *dentivalvata* species group of the genus *Meleonoma* Meyrick are described. They are *M. anisomorpha***sp. nov**., *M. apicitriangula***sp. nov**., *M. bisecta***sp. nov**., *M. corniculata***sp. nov**., *M. longisacca***sp. nov**., *M. ovaliuncatus***sp. nov**., *M. plicativa***sp. nov**., *M. producta***sp. nov**., *M. quadrata***sp. nov**., *M. renaria***sp. nov**., *M. simililunformis***sp. nov**., *M. strena***sp. nov**., and *M. utricularis***sp. nov**. Images of both adults and genitalia of the new species are provided.

## Introduction

*Meleonoma* Meyrick, 1914 belongs to the tribe Meleonomini of the subfamily Periacminae in the family Autostichidae (Wang and Li 2020), distributed in the Palearctic and Oriental regions. Most *Meleonoma* species in China were assigned into nine species groups (Wang et al. 2020; Zhu and Wang 2022).

The *dentivalvata* species group includes 21 described species to date (Wang et al. 2020, 2021). Members of the *dentivalvata* species group are defined by the broad dark lanceolate forewing with a yellow median costal spot and a yellow distal spot, or the median costal spot represented by a wide or lineate yellow median fascia, and the costal part of the valva usually with one or two apical or preapical spines in the male genitalia, and the ductus bursae sclerotised entirely or partly, and the corpus bursae with one or two signa or lacking a signum in the female genitalia.

The aim of the present paper is to describe 13 new species of the *dentivalvata* species group.

## Materials and methods

Specimens were collected by light traps in the mountainous areas of China, with permission of the local official government. Dissection and slide mounting of genitalia followed the methods introduced by Li (2002). Images of adults and genitalia were taken with Leica M205A and Leica DM750 microscopes, respectively, coupled with a Leica Application Suite 4.2 software.

Terminology follows Wang et al. (2020). Species are arranged in alphabetic order. All the studied specimens, including the types, are deposited in the Insect Collection of Nankai University, Tianjin, China (**NKU**).

## Taxonomy

***Meleonoma* Meyrick, 1914**

*Meleonoma* Meyrick, 1914: 255. Type species: *Cryptolechia stomota* Meyrick, 1910, by original designation.

*Acryptolechia* Lvovsky, 2010: 255. Type species: *Cryptolechia malacobyrsa* Meyrick, 1921, by original designation.

### Key to *Meleonoma* species of the *dentivalvata* group in China

**Table:** 

1	Forewing with a median costal spot and a distal spot	**2**
–	Forewing with a median fascia and a distal spot	**14**
2	Transtilla with a large dorsal spine at each side directed obliquely upward (Wang 2003: fig. 11)	***M. paranthaedeaga***
–	Transtilla without a dorsal spine	**3**
3	Tip of costa extended outward (Wang 2006a: fig. 5)	***M. rectimarginalis***
–	Tip of costa not extended outward apically	**4**
4	Costal part of valva with a narrow dentate plate running from near base to ~ 2/5 above ventral margin (Wang 2003: fig. 21)	***M. anthaedeaga***
–	Costal part of valva without such a dentate plate	**5**
5	Costal part of valva with three or four spines (Fig. 23)	***M. renaria* sp. nov**.
–	Costal part of valva with a spine	**6**
6	Costal part of valva with a few tiny teeth (Wang et al. 2021: fig. 22)	***M. globoidea***
–	Costal part of valva without tiny teeth	**7**
7	Costa with a small incision at base (Fig. 16)	***M. bisecta* sp. nov**.
–	Costa without incision at base	**8**
8	Sacculus elliptically dilated distally (Wang 2003: fig. 7)	***M. zhengi***
–	Sacculus not dilated distally	**9**
9	Saccus approximately as long as uncus	**10**
–	Saccus longer than uncus	**11**
10	Cornuti consisting of a pile of short spines and a large spine with several denticles (Wang 2003: fig. 5)	***M. falsivespertina***
–	Cornuti consisting of a pile of spines (Wang 2003: fig. 23)	***M. gei***
11	Sacculus triangular	**12**
–	Sacculus quadrate or trapezoidal	**13**
12	Aedeagus ~2× length of costal part of valva (Fig. 18)	***M. longisacca* sp. nov**.
–	Aedeagus slightly longer than costal part of valva (Fig. 26)	***M. utricularis* sp. nov**.
13	Sacculus quadrate (Fig. 20)	***M. plicativa* sp. nov**.
–	Sacculus trapezoidal (Fig. 22)	***M. quadrata* sp. nov**.
14	Forewing with a lineate median fascia (Wang 2003: fig. 1)	***M. deflecta***
–	Forewing with a broadly banded median fascia	**15**
15	Median fascia extending obliquely outward to above fold (Fig. 6)	***M. ovaliuncatus* sp. nov**.
–	Median fascia extending obliquely outward at end of fold	**16**
16	Costal part of valva with a large carina bifurcate distally (Wang 2004: fig. 6)	***M. furcellata***
–	Costal part of valva without such a carina	**17**
17	Sacculus concave medially at apex, forming two apical lobes (Wang 2006a: fig. 13)	***M. similifloralis***
–	Sacculus narrowed distally, not concave medially at apex	**18**
18	Costal part of valva concave inward at apex or concave near apex ventrally	**19**
–	Costal part of valva neither concave inward apically nor concave near apex ventrally	**22**
19	Costal part of valva concave inward at apex	**20**
–	Costal part of valva concave near apex ventrally	**21**
20	Sacculus with a clavate process (Fig. 24)	***M. simililunformis* sp. nov**.
–	Sacculus with a hook-shaped process distally (Wang 2006a: fig. 7)	***M. proximihamatilis***
21	Costal part of valva with a shallow subrectangular apical emargination (Wang 2003: fig. 24)	***M. varifascirupta***
–	Costal part of valva with a deep semi-circular apical emargination (Wang 2003: fig. 13)	***M. fascirupta***
22	Sacculus with an apical spine directed obliquely dorsad (Wang 2006a: fig. 4)	***M. luniformis***
–	Sacculus without an apical spine	**23**
23	Sacculus with a clavate ventral process (Fig. 14)	***M. anisomorpha* sp. nov**.
–	Sacculus without such a ventral process	**24**
24	Ventral margin of costal part of valva with a preapical spine	**25**
–	Ventral margin of costal part of valva without a preapical spine	**26**
25	Costal part of valva with a spine at apex (Fig. 15)	***M. apicitriangula* sp. nov**.
–	Costal part of valva without a spine at apex (Wang et al. 2021: fig. 24)	***M. proapicalis***
26	Costal part of valva with a ventroapical spine or a ventroapical denticle	**27**
–	Costal part of valva with an apical spine above ventral angle	**33**
27	Costal part of valva with a ventroapical denticle	**28**
–	Costal part of valva with a ventroapical spine	**29**
28	Costal part of valva with a spine above ventral angle (Wang et al. 2021: fig. 26)	***M. ventridentata***
–	Costal part of valva without a spine above ventral angle (Fig. 17)	***M. corniculata* sp. nov**.
29	Costal part of valva with a long ridge above ventral margin, serrate on ventral edge (Wang et al. 2021: fig. 25)	***M. raphidacantha***
–	Costal part of valva without such a ridge	**30**
30	Costal part of valva with several denticles apically (Fig. 21)	***M. producta* sp. nov**.
–	Costal part of valva without denticle apically	**31**
31	Corpus bursae shorter than ductus bursae (Fig. 25)	***M. strena* sp. nov**.
–	Corpus bursae longer than ductus bursae	**32**
32	Cornuti being a row of spines (Wang 2006b: fig. 256)	***M. torophanes***
–	Cornutus being a large spine (Wang 2006b: fig. 224)	***M. menglana***
33	Aedeagus curved in S shape in distal 1/2 (Wang et al. 2021: fig. 21)	***M. curvativa***
–	Aedeagus not distinctly curved distally (Wang et al. 2021: fig. 23)	***M. lunata***

#### Meleonoma anisomorpha sp. nov.

Taxon classificationAnimaliaLepidopteraAutostichidae

93EFBC45-A0D9-538A-B779-2B8652E53334

https://zoobank.org/01849DEE-93D7-4069-81DF-F6D46830A5E5

Figs 1, 14

##### Type material.

***Holotype*: China** • ♂; Xizang Autonomous Region [Tibet], Motuo County [Mêdog], Beibeng Town, Gelin Village; 29.25°N, 95.19°E; 1063 m a.s.l.; 29 VII.2018; Mujie Qi leg.; slide no. ZXJ19105, in NKU. ***Paratypes***: • 2 ♂; same data as for holotype; slide no. ZXJ19512, in NKU 1 ♂; Xizang Autonomous Region [Tibet], Motuo County [Mêdog], Beibeng Town; 810 m a.s.l.; 13 VIII.2017; Mujie Qi & Xiaofei Yang leg.; slide no. ZXJ19104, in NKU.

**Figures 1–8.**
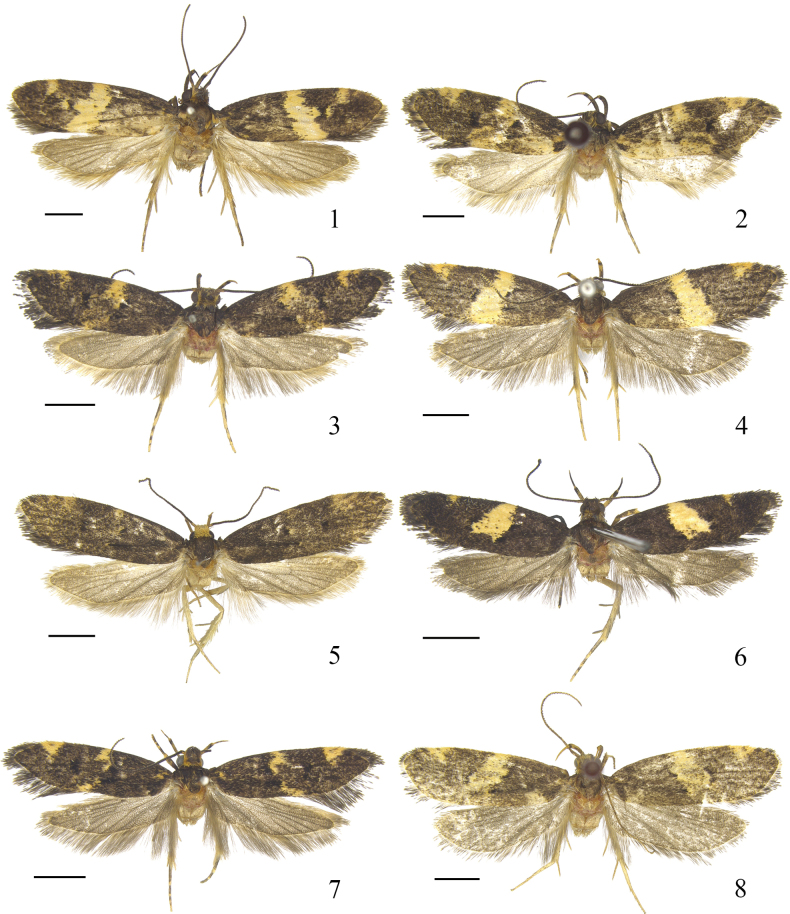
Dorsal habitus of *Meleonoma* spp. **1**. *M. anisomorpha* sp. nov., holotype, male, ZXJ19105; **2**. *M. apicitriangula* sp. nov., holotype, male, ZXJ19482; **3**. *M. bisecta* sp. nov., holotype, male, ZXJ19514; **4**. *M. corniculata* sp. nov., holotype, male, ZXJ19092; **5**. *M. longisacca* sp. nov., paratype, male, ZXJ19488; **6**. *M. ovaliuncatus* sp. nov., holotype, male, ZXJ19513; **7**. *M. plicativa* sp. nov., holotype, male, ZXJ19540; **8**. *M. producta* sp. nov., holotype, male, ZXJ19097. Scale bars: 2.0 mm.

**Figures 9–13.**
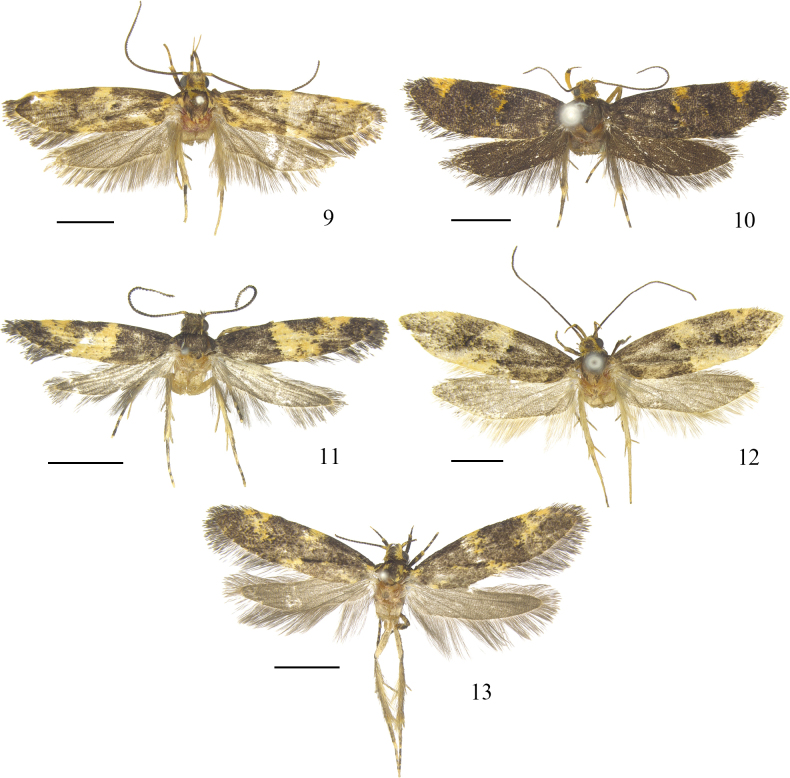
Dorsal habitus of *Meleonoma* spp. **9**. *M. quadrata* sp. nov., holotype, male, ZXJ19538; **10**. *M. renaria* sp. nov., holotype, male, ZXJ19520; **11**. *M. simililunformis* sp. nov., holotype, male, ZXJ19633; **12**. *M. strena* sp. nov., paratype, female, ZXJ19534; **13**. *M. utricularis* sp. nov., paratype, female, ZXJ19530. Scale bars: 2.0 mm.

**Figures 14–19.**
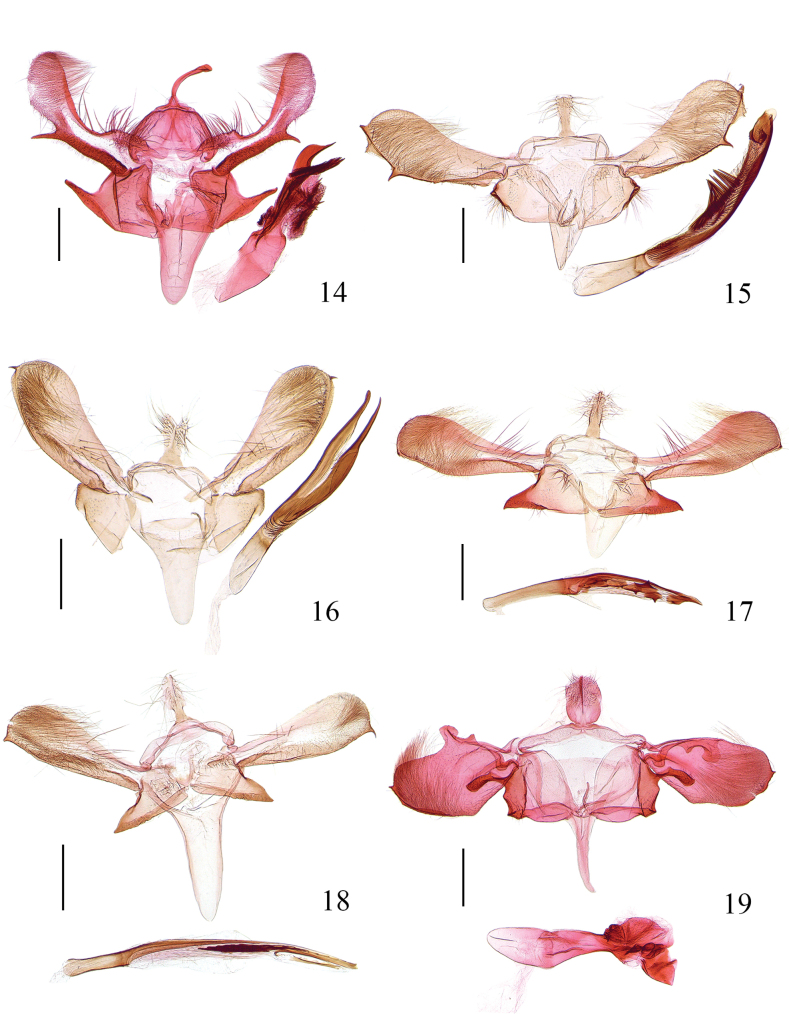
Male genitalia of *Meleonoma* spp. **14**. *M. anisomorpha* sp. nov., holotype, ZXJ19105; **15**. *M. apicitriangula* sp. nov., holotype, ZXJ19482; **16**. *M. bisecta* sp. nov., holotype, ZXJ19514; **17**. *M. corniculata* sp. nov., holotype, ZXJ19092; **18**. *M. longisacca* sp. nov., holotype, ZXJ19487; **19**. *M. ovaliuncatus* sp. nov., paratype, ZXJ19119. Scale bars: 0.5 mm.

##### Diagnosis.

The new species is similar to *M. apicitriangula* sp. nov. in the male genitalia by the ventral margin of the costal part of the valva with a preapical spine. It can be distinguished by the costal band rectangularly produced basally, the costal part of the valva without an apical spine, and the sacculus with a clavate ventroapical process. In *M. apicitriangula*, the costa is not rectangularly produced basally, the costal part of the valva has an apical spine, and the apex of the sacculus lacks a clavate process.

##### Description.

Forewing length 7.0–7.5 mm (Fig. 1).

***Head*** yellowish brown, yellow laterally on occiput. Labial palpus black; second segment mixed with yellow scales on inner and dorsal surfaces, with a yellow ring at apex; third segment shorter than second segment, yellow at apex. Antenna black.

***Thorax*** black, yellowish laterally; tegula black, yellowish apically. Forewing with costal margin arched, apex rounded; ground colour black; with a whitish yellow spot at base above fold, different in size within individuals; median fascia whitish yellow, extending from before middle of costal margin obliquely outward to before tornus, widened posteriorly; distal spot placed at distal 1/4, narrowed posteriorly; plical spot black, placed at inner margin of median fascia, not clearly distinguished from ground colour; discal and discocellular spots black, placed at inner and outer margins of median fascia, not clearly distinguished from ground colour, discocellular spot with a whitish yellow spot on outer side; dorsum with a whitish yellow spot at base; fringe yellowish brown, with a yellow basal line. Hindwing and fringe brown.

***Legs*** whitish yellow, with exceptions on ventral surface: foreleg dark brown, tarsus yellow at apices of basal two tarsomeres, midleg with femur dark brown apically, tibia dark brown, yellow at middle and at apex, respectively, tarsus dark brown, yellow at apices of basal three tarsomeres and yellow at apical tarsomere, hindleg with tibia scattered with dark brown scales, tarsus with basal four tarsomeres dark brown, yellow at apices.

***Abdomen***: Male genitalia (Fig. 14). Uncus clavate, dilated at apex. Tegumen with lateral arm narrowed anteriorly, forming a V-shaped anterior emargination. Costal part of valva nearly uniform in width in basal 1/2, ovately dilated in distal 1/2; ventral margin heavily sclerotised from base to beyond middle, forming a band ending with a stout spine, different in size in left and right costal parts of valvae; costal band rectangularly produced basally, narrowly banded distally, reaching before tip, concave before middle, gently arched distally, with long setae; transtilla widened to middle, blunt at apex. Sacculus subquadrate in basal 2/3, distal 1/3 produced to a clavate ventral process. Saccus ~ 1.5× length of uncus, wide at base, narrowed from base to rounded apex. Juxta U-shaped. Aedeagus slightly longer than costal part of valva, tubular in basal 2/5, distal 3/5 produced to two sclerotised bands: the longer one smooth, pointed at apex, the shorter one densely with spines; cornuti composed of fine spines, placed in vesica.

Female unknown.

##### Etymology.

The specific epithet is derived from the Greek *aniso*- and -*morphus*, referring to the stout spine on the ventral margin of the costal parts of the left and right valvae differently sized.

##### Distribution.

China (Xizang Autonomous Region [Tibet]).

#### Meleonoma apicitriangula sp. nov.

Taxon classificationAnimaliaLepidopteraAutostichidae

FCC7AFE1-AAEB-5FAC-9DC4-6E036FE77EB6

https://zoobank.org/9D787801-3D2D-41D1-8369-C9D779AE4A7D

Figs 2, 15, 27

##### Type material.

***Holotype*: China** • ♂; Xizang Autonomous Region [Tibet], Bomi County, Tongmai Town; 30.10°N, 95.08°E; 2029 m a.s.l.; 12 VII.2018; Mujie Qi leg.; slide no. ZXJ19482, in NKU. ***Paratypes***: • 3 ♀; same data as for holotype; slide nos. ZXJ19506, ZXJ19629, in NKU 7 ♂ 8 ♀; 13–16 VII.2018; other data same as for holotype, in NKU 2 ♂ 5 ♀; • Xizang Autonomous Region [Tibet], Motuo County [Mêdog]; 2089 m a.s.l.; 6–19 VIII.2017; Mujie Qi & Xiaofei Yang leg.; slide no. ZXJ19483 ♂, in NKU 5 ♂ 4 ♀; • Xizang Autonomous Region [Tibet], Motuo County [Mêdog]; 2076 m; 28 VII–9 VIII.2018; Mujie Qi leg.; slide no. ZXJ19484 ♂, in NKU 3 ♂ 3 ♀; • Xizang Autonomous Region [Tibet], Pailong; 2031 m a.s.l.; 18 VIII.2018; Mujie Qi leg.; slide no. ZXJ19485, in NKU 2 ♀, • Xizang Autonomous Region [Tibet], nielamu County, Zhangmu Town; 1961 m a.s.l.; 5–8 VII.2019; Mujie Qi & Jiaqi Deng leg.; in NKU.

**Figures 20–26.**
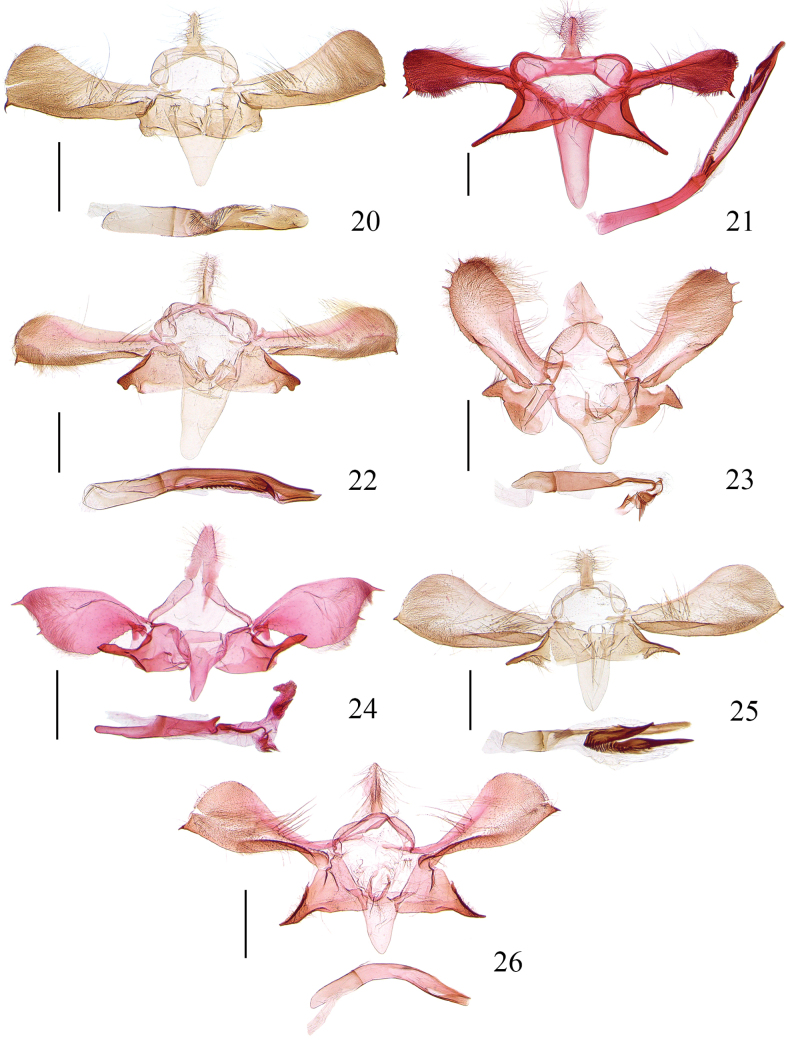
Male genitalia of *Meleonoma* spp. **20**. *M. plicativa* sp. nov., holotype, ZXJ19540; **21**. *M. producta* sp. nov., paratype, ZXJ19103; **22**. *M. quadrata* sp. nov., holotype, ZXJ19538; **23**. *M. renaria* sp. nov., holotype, ZXJ19520; **24**. *M. simililunformis* sp. nov., holotype, ZXJ19633; **25**. *M. strena* sp. nov., holotype, ZXJ19526; **26**. *M. utricularis* sp. nov., holotype, ZXJ19490. Scale bars: 0.5 mm.

**Figures 27–33.**
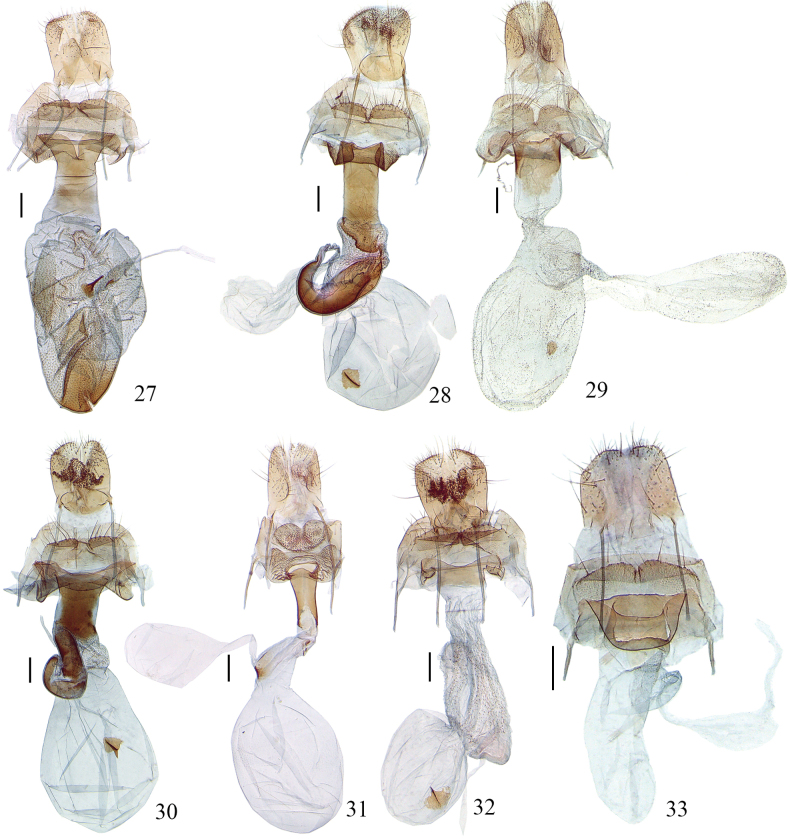
Female genitalia of *Meleonoma* spp. **27**. *M. apicitriangula* sp. nov., paratype, ZXJ19506; **28**. *M. longisacca* sp. nov., paratype, ZXJ19508; **29**. *M. ovaliuncatus* sp. nov., paratype, ZXJ19619; **30**. *M. producta* sp. nov. paratype, ZXJ19510; **31**. *M. renaria* sp. nov., paratype, ZXJ19533; **32**. *M. strena* sp. nov., paratype, ZXJ19534; **33**. *M. utricularis* sp. nov., paratype, ZXJ19509. Scale bars: 0.2 mm.

##### Diagnosis.

The new species is similar to *M. proapicalis* Wang, 2021 (Wang, Zhu and Tao 2021) in male genitalia. It can be distinguished by the costal part of the valva with a spine at the ventral angle, and with a triangular plate before apex on the ventral margin in the male genitalia, and the spine-shaped signum in the female genitalia. In *M. proapicalis*, the costal part of the valva lacks a spine at the ventral angle, lacks a plate before apex on the ventral margin, and the signum is narrowly elongate. It is also similar to *M. anisomorpha* sp. nov., and the differences between them can be found in the diagnosis of *M. anisomorpha* sp. nov.

##### Description.

Forewing length 7.5–8.5 mm (Fig. 2).

***Head*** with frons dark brown, vertex pale yellow, occiput pale yellow mixed with dark brown. Labial palpus dark brown; second segment with a yellow dot distally on inner surface, forming a yellow ring at apex; third segment ~ 1/2 length of second segment, yellow on dorsal and inner surfaces. Antenna dark brown; scape yellow at apex; flagellum alternated with whitish yellow on ventral surface.

***Thorax*** and tegula dark brown. Forewing with costal margin arched, apex rounded; dark brown, with a yellow spot at base; median fascia yellow, with scattered greyish brown scales, from before middle of costal margin extending obliquely outward to before tornus, narrower anteriorly; distal spot yellow, with scattered greyish brown scales, inverted triangular, placed beyond distal 1/3; dorsum with yellow scales basally, forming a spot; plical spot black, at middle of fold, placed at inner margin of median fascia; discal and discocellular spots black, discal spot ovate, placed at inner margin of median fascia, discocellular spot larger, banded, placed at outer margin of median fascia; fringe dark brown, with a yellowish white basal line. Hindwing and fringe brown.

***Legs*** yellowish white, with exceptions on ventral surface: coxa and femur of foreleg brown, femora of mid- and hindlegs with scattered brown scales, tibiae of fore- and midlegs dark brown except yellow at middle and at apex, tibia of hindleg with scattered dark brown scales, tarsus of foreleg dark brown except yellow at apices of basal two tarsomeres, tarsus of midleg dark brown except yellow at apices of basal two tarsomeres and yellow at apical tarsomere, tarsus of hindleg with basal three tarsomeres dark brown except yellow at base of basal tarsomere and at apices.

***Abdomen***: Male genitalia (Fig. 15). Uncus wide at base, narrowed near base, then widened from near base to apex, with dense long setae distally. Tegumen narrowed medially; lateral arm uniform, blunt at apex. Costal part of valva narrow at base, gradually widened to basal 2/3, thereafter narrowed to apex; apex obtuse, with a spine at ventral angle; ventral margin heavily sclerotised, folded dorsad basally, forming two layers, dorsal layer dentate distally, with a preapical spine, placed at basal 2/3, with a triangular plate before apex, dentated distally, ventral layer short, dentated in distal 3/4; costa produced distally; transtilla clavate. Sacculus triangular; dorsal margin with a band, folded ventrad, sinuate; ventral margin folded dorsad and lined with long setae in distal 2/5; with a setose plate at base. Saccus broad, ~2× length of uncus. Juxta U-shaped. Aedeagus ~2× length of costal part of valva, tubular in basal 1/3, distal 2/3 narrower, with fine wrinkles, with a process before apex, densely with tiny spines; cornuti being a row of spines, placed at beyond middle.

Female genitalia (Fig. 27). Papillae anales subquadrate, with sparse setae dorsally. Apophyses posteriores ~3× length of apophyses anteriores. Eighth tergite subtrapezoidal. Eighth sternal plate spiculate; with a median groove, laterally with a triangular plate, narrowed to posterolateral corner, lined with long setae on posterior margin. Antrum trapezoidal, deeply concave medially on ventral surface, obtuse on inner margin. Ductus bursae weakly sclerotised, membranous anteriorly. Corpus bursae large, with an ovate plate anteriorly; ductus seminalis arising from middle of corpus bursae; signum long spine-shaped, with a subovate base.

##### Distribution.

China (Xizang Autonomous Region [Tibet]).

##### Etymology.

The specific epithet is derived from the Latin *apic*- and *triangulus*, referring to the costal part of the valva with a triangular plate before apex in the male genitalia.

#### Meleonoma bisecta sp. nov.

Taxon classificationAnimaliaLepidopteraAutostichidae

FCDB6A90-8D7D-5B4F-8A86-8BEF9BD3F9A4

https://zoobank.org/FB9B44CC-F1E5-4805-9648-4C726C1E6DC0

Figs 3, 16

##### Type material.

***Holotype*: China** • ♂; Xizang Autonomous Region [Tibet], Motuo County [Mêdog], Beibeng Town; 29.24°N, 95.17°E; 987 m a.s.l.; 12 VIII.2017; Mujie Qi & Xiaofei Yang leg.; slide no. ZXJ19514, in NKU. ***Paratypes***: • 7 ♂; 13–15 VIII.2017; other data same as for holotype, in NKU 1 ♂; • Xizang Autonomous Region [Tibet], Motuo County [Mêdog], Beibeng Town, Jiangxin Village; 780 m a.s.l.; 30 VII.2018; Mujie Qi leg.; slide no. ZXJ19491, in NKU.

##### Diagnosis.

The new species is similar to *M. plicativa* sp. nov. in both forewing pattern and male genitalia. It can be distinguished by the uncus concave medio-apically, the triangular sacculus and the aedeagus bilobed in distal 2/3. In *M. plicativa*, the uncus is not concave medio-apically, the sacculus is quadrate, and the aedeagus is not bilobed in distal 2/3.

##### Description.

Forewing length 5.5–7.0 mm (Fig. 3).

***Head*** yellowish, tipped with black on vertex and on occiput. Labial palpus with first and second segments black on outer surface, yellow on inner surface; second segment with scattered black scales on inner surface, forming a black ring before apex; third segment with scattered black scales on outer surface. Antenna black; scape yellow apically on ventral surface; flagellum alternated with yellow on ventral surface.

***Thorax*** and tegula black. Forewing black, tinged with yellow scales; median costal spot yellow, situated before middle, widened posteriorly; distal spot yellow, at distal ~1/4, mixed with black scales; plical spot black, at beyond middle of fold, with yellow scales on its outer side; discal and discocellular spots black, placed at basal 3/5 and at outer margin of cell, respectively; fringe black, with a yellow basal line. Hindwing and fringe brown.

***Legs*** yellow, with exceptions on ventral surface: coxa of foreleg with scattered black scales, femur and tibia black except yellow apically, tarsus black except yellow at base of basal tarsomere and at apices of basal two and apical one tarsomeres, femur of midleg mixed with black scales, tibia black except yellow at middle and at apex, tarsus black except yellow at apices of basal two tarsomeres, tibia of hindleg with scattered black scales, tarsus with basal four tarsomeres black except yellow at base of basal tarsomere and at apices.

***Abdomen***: Male genitalia (Fig. 16). Uncus subrectangular, concave medio-apically, setose in distal 2/3. Tegumen narrowed medially; lateral arm widened medially, narrowed anteriorly to rounded apex. Costal part of valva narrow at base, slightly widened to basal 2/3, distal 1/3 narrowed, with sparse long setae medially, with fine hairs ventrodistally; apex rounded, with a spine at ventral angle; ventral margin produced medially; costa with a small incision at base, produced distally; transtilla short, blunt at apex. Sacculus triangular, wide at base, gradually narrowed to rounded apex; ventral margin folded dorsad basally, subtriangularly produced at pre-apex. Saccus ~ 1.5× length of uncus, narrowed from wide base to narrowly rounded apex. Juxta slender, U-shaped. Aedeagus 1.5× length of costal part of valva, tubular basally, with fine wrinkles at basal 1/3, bilobed in distal 2/3: dorsal lobe wider in basal 3/4, slender in distal 1/4, rounded at apex, ventral lobe wider in basal 2/3, spine-shaped in distal 1/3, pointed at apex.

Female unknown.

##### Etymology.

The specific epithet is derived from the Latin *bisectus*, referring to the distally bilobed aedeagus.

##### Distribution.

China (Xizang Autonomous Region [Tibet]).

#### Meleonoma corniculata sp. nov.

Taxon classificationAnimaliaLepidopteraAutostichidae

8BD40620-D89B-51FF-A034-81BBDB9E4251

https://zoobank.org/EAEFA940-5545-4A6B-AD38-6A166BB5FD23

Figs 4, 17

##### Type material.

***Holotype*: China** • ♂; Sichuan Prov., Chengdu, Anzi River, Baliping; 30.77°N, 103.22°E; 1706 m a.s.l.; 24 VI.2016; Kaijian Teng & Xiaofei Yang leg.; slide no. ZXJ19092, in NKU. ***Paratypes***: • 2 ♂; 25 VI.2016; other data same as for holotype; slide no. ZXJ19344, in NKU 1 ♂; • Sichuan Prov., Xiaojin County, Mt. Sigu’niang; 3243 m a.s.l.; 4 VII.2016; Kaijian Teng & Xiaofei Yang leg.; slide no. ZXJ19094, in NKU.

##### Diagnosis.

The new species is similar to *M. longisacca* sp. nov. in male genitalia. It can be distinguished by the forewing with a median fascia, the saccus 1.5× the length of the uncus, and the cornuti being several sclerites, each of which a large tooth in the male genitalia. In *M. longisacca*, the forewing lacks a median fascia, but represented by a median costal spot, the saccus is 2.5× the length of the uncus, and the cornuti consist of a pile of spines.

##### Description.

Forewing length 7.0–7.5 mm (Fig. 4).

***Head*** yellow, tipped with dark brown on occiput. Labial palpus yellow; first and second segments scattered with dark brown scales on outer surface; second segment forming an indistinct ring at apex; third segment 2/3 length of second segment. Antenna dark brown on dorsal surface, yellow on ventral surface; scape yellow apically on dorsal surface; flagellum alternated with yellow in distal 1/3 on dorsal surface.

***Thorax*** and tegula black. Forewing with costal margin arched, apex rounded; ground colour dark brown; median fascia yellow, extending from before middle of costal margin obliquely outward to before tornus, widened posteriorly; distal spot yellow, triangular, at distal 1/4, narrowed posteriorly, with sparse dark brown scales; plical spot black, placed at distal 2/5 of fold, not clearly distinguished from ground colour; discal and discocellular spots black, placed at basal 3/5 and at outer margin of cell, respectively, the latter larger, banded; fringe dark brown, with a yellowish white basal line. Hindwing and fringe brown.

***Legs*** yellow, with exceptions on ventral surface: foreleg dark brown except coxa yellow apically, tarsus yellow at base of basal tarsomere and at apices of basal two tarsomeres and yellow at apical tarsomere, femur of midleg with dark brown scales at base and at apex, tibia dark brown except yellow at middle and at apex, tarsus dark brown except yellow at apices of basal two and apical one tarsomeres, tibia of hindleg with scattered dark brown scales, tarsus with basal four tarsomeres dark brown except yellow at apices.

***Abdomen***: Male genitalia (Fig. 17). Uncus lanceolate, with basal 1/3 uniformly wide, concave at beyond basal 1/3 and widened to basal 1/2, thereafter narrowed to narrowly rounded apex, setose laterally. Tegumen uniform in width medially; lateral arm narrowed to apex. Costal part of valva narrow at base, widened from base to basal 2/3, distal 1/3 uniformly wide except slightly narrower apex, rounded at apex, with several stout setae before middle, with fine hairs distally; apex rounded, with a denticle at ventral angle; ventral margin gently produced medially, with a sclerotised carina extending from base to basal 1/2 reaching at median width of costal part of valva, with spare setae; costal band concave basally, then gently arched distally; transtilla subovate. Sacculus triangular, weakly sclerotised in basal 2/3, distal 1/3 heavily sclerotised, with a small process at apex, with tufted setae basally. Saccus 1.5× length of uncus, wide at base, narrowed from base to rounded apex. Juxta weakly sclerotised, U-shaped. Aedeagus tubular in basal 2/5, distal 3/5 narrowly banded; cornuti being several sclerites, each with a large tooth.

Female unknown.

##### Etymology.

The specific epithet is from the Latin *corniculatus*, referring to the cornuti in the male genitalia.

##### Distribution.

China (Sichuan Province).

#### Meleonoma longisacca sp. nov.

Taxon classificationAnimaliaLepidopteraAutostichidae

96F38679-B885-5902-A813-D0798A33D6A4

https://zoobank.org/9ECDAB97-042F-4D81-B5B0-06C9E93B6B02

Figs 5, 18, 28

##### Type material.

***Holotype*: China** • ♂; Xizang Autonomous Region [Tibet], Jilong County, Jilong Town, Chongdui Village; 28.41°N, 85.31°E; 2858 m a.s.l.; 13 VII.2019; Mujie Qi & Jiaqi Deng leg.; slide no. ZXJ19487, in NKU. ***Paratypes***: • 4 ♂ 3 ♀; same data as for holotype; slide no. ZXJ19508 ♀, in NKU 4 ♂ 3 ♀; 12–15 VII.2019; other data same as for holotype; slide no. ZXJ19488 ♂, in NKU.

##### Diagnosis.

The new species is similar to *M. torophanes* (Meyrick, 1935) in male genitalia. It can be distinguished by the forewing with a median costal spot, the saccus ~ 2.5× the length of the uncus in the male genitalia; the quadrate signum with a mesial ridge in the female genitalia. In *M. torophanes*, the forewing lacks a median costal spot, but represented by a median fascia (Wang 2006b: 152, fig. 130), the saccus is slightly longer than the uncus (Wang 2006b: 152, fig. 256); and the ovate signum has several denticles and an apical spine (Wang 2006b: 152, fig. 257). It is also similar to *M. utricularis* sp. nov. and *M. corniculata* sp. nov., and the differences between them can be found in the diagnosis of the latter two species.

##### Description.

Forewing length 7.0–7.5 mm (Fig. 5).

***Head*** pale yellow. Labial palpus pale yellow, with sparse dark brown scales on second segment; third segment ~1/2 length of second segment. Antenna dark brown; scape mixed with yellow scales.

***Thorax*** and tegula dark brown. Forewing dark brown, tinged with yellowish brown scales; median costal spot yellowish brown, situated at before middle; distal spot yellowish brown, with scattered dark brown scales, at distal ~1/4; plical spot black, not clearly distinguished from ground colour, with a yellowish brown spot on its outer side; discal and discocellular spots black, the former with a yellowish brown spot on its outer side; fringe greyish black, with a yellowish brown basal line. Hindwing and fringe brown.

***Legs*** yellow, with exceptions on ventral surface: coxa of foreleg with scattered dark brown scales, femur dark brown, femur of midleg dark brown apically, tibiae of fore- and midlegs dark brown except yellow at middle and at apex, tibia of hindleg with scattered dark brown scales, tarsus of foreleg dark brown except yellow at apex of basal tarsomere, tarsus of midleg dark brown except yellow at apex of basal tarsomere and yellow at apical tarsomere, tarsus of hindleg with scattered dark brown scales on basal three tarsomeres, denser at basal tarsomere.

***Abdomen***: Male genitalia (Fig. 18). Uncus with basal 1/3 rectangular, distal 2/3 lanceolate, setose distally. Tegumen uniform, banded. Costal part of valva narrow at base, widened to basal 1/2, almost uniformly wide in distal 1/2 except slightly narrower apex, with fine hairs distally; ventral margin heavily sclerotised, straight basally, slightly produced beyond middle, ending with a spine; costal band reaching before middle; transtilla slenderly clavate. Sacculus triangular, narrowed from wide base to pointed apex, heavily sclerotised on dorsal and ventral margins. Saccus elongate, ~ 2.5× length of uncus, narrowed from base to narrowly rounded apex. Juxta weakly sclerotised, V-shaped. Aedeagus straight, ~2× length of costal part of valva, tubular in basal 1/4, then narrowed to apex, apex extended towards the base, forming a slender bar, dentated dorsally, below it with a slender band distally; cornuti being a pile of spines, ranging from middle to beyond basal 3/4.

Female genitalia (Fig. 28). Papillae anales subquadrate. Apophyses anteriores ~ 1/3 as long as apophyses posteriores. Eighth sternal plate with a narrow median groove, laterally with a semi-ovate plate, lined with long setae on posterior margin, straight on anterior margin. Lamella antevaginalis subrectangular, with lateral sides folded dorsad. Ductus bursae heavily sclerotised in posterior 2/3, with tiny teeth at posterior 2/3 dorsoanteriorly, membranous in anterior 1/3; appendix bursae arising from junction of ductus bursae and corpus bursae, heavily sclerotised. Corpus bursae approximately as long as ductus bursae, rounded; signum quadrate, with a ridge mesially.

##### Etymology.

The specific epithet is derived from the Latin *longi*- and the term *saccus*, referring to the elongate saccus.

##### Distribution.

China (Xizang Autonomous Region [Tibet]).

#### Meleonoma ovaliuncatus sp. nov.

Taxon classificationAnimaliaLepidopteraAutostichidae

1EE6D15A-8BAB-58EC-AC5A-BEE24B04B156

https://zoobank.org/921781AF-AE10-4870-9FD5-36C06C14F103

Figs 6, 19, 29

##### Type material.

***Holotype*: China** • ♂; Xizang Autonomous Region [Tibet], Motuo County [Mêdog], Yadong Village; 29.33°N, 95.34°E; 833 m a.s.l.; 16 VIII.2017; Mujie Qi & Xiaofei Yang leg.; slide no. ZXJ19513, in NKU. ***Paratypes***: • 24 ♂ 10 ♀; same data as for holotype; slide nos. ZXJ19116 ♂, ZXJ19117 ♀, ZXJ19619 ♀, in NKU 17 ♂ 1 ♀; 12–15 VIII.2017; other data same as for holotype; slide nos. ZXJ19119 ♂, ZXJ19515 ♂, in NKU 8 ♂; • Xizang Autonomous Region [Tibet], Motuo County [Mêdog], Dexing Village; 833 m a.s.l.; 18 VIII.2017; Mujie Qi & Xiaofei Yang leg.; slide no. ZXJ19118, in NKU.

##### Diagnosis.

The new species is similar to *M. gei* (Wang, 2003) in the forewing pattern. It can be distinguished in the male genitalia by the ovate uncus, the costal part of the valva with a subrectangular process at base and the aedeagus with several sclerites distally. In *M. gei*, the uncus is rectangular, the costal part of the valva lacks a process at base and the aedeagus has a pile of spines instead of sclerites distally (Wang 2003: 210, fig. 23).

##### Description.

Forewing length 5.0–6.0 mm (Fig. 6).

***Head*** black, yellow on frons. Labial palpus yellow; first segment black on outer surface; second segment with scattered black scales in distal 1/2, forming a black ring at apex; third segment black except yellow at base and at apex on dorsal surface. Antenna black except scape yellow on posterior margin.

***Thorax*** and tegula black. Forewing with costal margin arched, apex rounded; ground colour black; median fascia yellow, extending from before middle of costal margin obliquely outward to above distal 1/3 of fold, widened posteriorly; distal spot small, yellow, placed at distal 1/4; plical, discal and discocellular spots black, not clearly distinguished from ground colour; fringe black. Hindwing and fringe dark grey.

***Legs*** yellow, with exceptions on ventral surface: coxa of foreleg with scattered dark brown scales, femur dark brown except yellow apically, tibia dark brown except yellow at middle and at apex, tarsus dark brown except yellow at apices of basal two tarsomeres, femora of mid- and hindlegs with scattered dark brown scales, tibiae of mid- and hindlegs dark brown except yellow apically, tarsus of midleg dark brown except yellow at apices of basal two and apical one tarsomeres, tarsus of hindleg with basal four tarsomeres dark brown except yellow at apices.

***Abdomen***: Male genitalia (Fig. 19). Uncus ovate, setose on dorsal surface. Tegumen narrowed medially; lateral arm uniform, blunt at apex. Costal part of valva foliaceous, roundly dilated, with fine hairs distally, with a subrectangular process at base, extending ventrad; apex with a spine above ventral angle; ventral margin with basal 2/5 straight, distal 3/5 gently produced; costal band with a triangular process near base, followed by a quadrate process; transtilla short, narrowed extending toward middle, pointed at apex. Sacculus subtrapezoidal, wide at base, narrowed from base to before apex; apex produced to a clavate process dorsally, slightly concave below it, forming an indistinct median process, rounded; ventral margin folded dorsad, with a triangular process apically, pointed at apex. Saccus elongate, clavate, ~2× length of uncus, rounded at apex. Juxta slender, U-shaped. Aedeagus slightly longer than costal part of valva, tubular in basal 1/2, sharply narrowed at beyond middle, with several sclerites distally; cornutus clavate, placed at middle of aedeagus.

Female genitalia (Fig. 29). Papillae anales rectangular, setose on dorsal surface. Apophyses posteriores ~ 2.5× length of apophyses anteriores. Eighth sternal plate deeply concave medially and line with long setae on posterior margin, distinctly produced medially on anterior margin. Lamella antevaginalis being two subovate plates, distributed on both sides of ductus bursae. Ductus bursae sclerotised in posterior 1/2, membranous in anterior 1/2. Corpus bursae ovate; appendix bursae membranous, arising from posterior part of corpus bursae; signum subrounded, dentate.

##### Etymology.

The specific epithet is derived from the Latin *oval*- and the term *uncus*, referring to the shape of the uncus.

##### Distribution.

China (Xizang Autonomous Region [Tibet]).

#### Meleonoma plicativa sp. nov.

Taxon classificationAnimaliaLepidopteraAutostichidae

8C19F792-D323-5ABA-90FE-5BBAADBFCCA7

https://zoobank.org/36AE8EB5-EE21-4053-8765-16400DF779BC

Figs 7, 20

##### Type material.

***Holotype*: China** • ♂; Yunnan Prov., Pu’er City, Taiyang River; 22.68°N, 101.03°E; 1450 m a.s.l.; 14 VIII.2016; Kaijian Teng et al. leg.; slide no. ZXJ19540, in NKU.

##### Diagnosis.

The new species can be distinguished by the sacculus with a line-shaped setose process near base and the aedeagus with fine wrinkles. It is similar to *M. bisecta* sp. nov., and the differences between them can be found in the diagnosis of *M. bisecta* sp. nov.

##### Description.

Forewing length 6.5 mm (Fig. 7).

***Head*** black, frons yellow, occiput yellow tipped with black laterally. Labial palpus yellow; first segment black on outer surface; second segment with scattered black scales on outer and dorsal surfaces, forming a black ring before apex; third segment with scattered black scales on outer and ventral surfaces, forming a black spot near base on dorsal surface. Antenna black; scape yellow apically on ventral surface; flagellum alternated with yellow on ventral surface.

***Thorax*** and tegula black. Forewing black, tinged with yellow scales, with a yellow stripe extending from near base above fold arched inward to reaching near base of fold; median costal spot yellow, situated at before middle, subrectangular; distal spot large, yellow, at distal ~1/3; plical spot black, not clearly distinguished from ground colour, at distal ~2/5 of fold, with a yellow spot on its outer side reaching end of fold; discal and discocellular spots black, placed at basal 3/5 and at outer margin of cell, respectively, the former with a yellow spot on its outer side, touching median costal spot; fringe dark brown, with a yellow basal line. Hindwing and fringe brown.

***Legs*** yellow, with exceptions on ventral surface: foreleg black except coxa and tibia yellow apically, tarsus yellow at base of basal tarsomere and at apices of basal two and apical one tarsomeres, femur of midleg black apically, tibia black except yellow at middle and at apex, tibia of hindleg with scattered dark brown scales, tarsi of mid- and hindlegs with basal four tarsomeres black except yellow at apices.

***Abdomen***: Male genitalia (Fig. 20). Uncus wide at base, narrowed to basal 1/3, lanceolate in distal 2/3, setose in distal 2/3. Tegumen narrowed medially; lateral arm uniform, blunt at apex. Costal part of valva narrow at base, gradually widened to rounded apex, with sparse long setae before middle, with fine hairs distally; ventral margin heavily sclerotised, gently produced, ending with a spine; costa concave medially, produced distally; transtilla short, narrowed toward middle, pointed at apex. Sacculus quadrate, wide at base, gradually narrowed to before apex, with a setose band basally, with a sclerotised band above ventral margin; apex straight in anterior 1/3, forming a setose process in posterior 2/3. Saccus longer than 1.5× length of uncus, wide at base, gradually narrowed from base to apex, narrowly rounded at apex. Juxta weakly sclerotised, V-shaped. Aedeagus slightly shorter than 1.5× length of costal part of valva, tubular in basal 2/5, with fine wrinkles from beyond basal 2/5 to distal 1/5, distal 3/5 partly sclerotised, rounded at apex.

Female unknown.

##### Etymology.

The specific epithet is derived from the Latin *plicativus*, referring to the fine wrinkles of the aedeagus.

##### Distribution.

China (Yunnan Province).

#### Meleonoma producta sp. nov.

Taxon classificationAnimaliaLepidopteraAutostichidae

DBAD148B-BBE1-5023-A259-34D0D2B0FD6B

https://zoobank.org/B217ACB0-384C-4367-8442-4684A97E7343

Figs 8, 21, 30

##### Type material.

***Holotype*: China** • **♂**; Sichuan Prov., Ya’an City, Bifengxia; 30.07°N, 102.97°E; 1115 m a.s.l.; 27 VI.2016; Kaijian Teng & Xiaofei Yang leg.; slide no. ZXJ19097, in NKU. ***Paratypes***: • 1 ♂ 1 ♀; same data as for holotype; slide nos. ZXJ19503 ♂, ZXJ19510 ♀, in NKU 1 ♂ 1 ♀; 28 VI.2016; other data same as for holotype; slide no. ZXJ19103 ♂, in NKU.

##### Diagnosis.

The new species is similar to *M. menglana* (Wang, 2006b) in male genitalia. It can be distinguished by the costal part of the valva bearing several denticles apically and the elongate triangular sacculus in the male genitalia, and the corpus bursae with one signum in the female genitalia; in *M. menglana*, the costal part of the valva lacks denticles apically, the sacculus is trapezoidal (Wang 2006b: 135, fig. 224), and the corpus bursae has two signa (Wang 2006b: 135, fig. 225). It is also similar to *M. strena* sp. nov., and the differences between them can be found in the diagnosis of the latter species.

##### Description.

Forewing length 7.0–8.0 mm (Fig. 8).

***Head*** greyish brown, whitish yellow on vertex, mixed with yellow on occiput. Labial palpus with first and second segments dark brown on outer surface, yellow on inner surface; second segment mixed with dark brown scales on inner surface, forming a dark brown ring at apex; third segment yellow, ~ 2/3 length of second segment. Antenna dark brown on dorsal surface, yellow on ventral surface; scape mixed with yellow on dorsal surface.

***Thorax*** and tegula dark brown. Forewing dark brown, with costal margin arched, apex rounded; a small yellow spot placed at base above fold; median fascia yellow, mixed with greyish brown scales, extending before middle of costal margin obliquely outward to before end of fold, nearly equal in width; distal spot yellow, mixed with greyish brown scales, inverted triangular, with a greyish brown dot anteriorly; plical spot black, at distal ~2/5 of fold, placed at inner margin of median fascia; discal and discocellular spots black, placed at inner and outer margins of median fascia, respectively; fringe dark brown, with a yellowish white basal line. Hindwing and fringe brown.

***Legs*** yellowish white, with exceptions on ventral surface: coxa and femur of foreleg with scattered brown scales, tibia brown, femur of midleg brown apically, tibia dark brown except yellow at middle and at apex, tarsi of fore- and hindlegs with sparse brown scales, hindleg with sparse brown scale on tibia.

***Abdomen***: Male genitalia (Fig. 21). Uncus wide at base, narrowed from base to before middle, thereafter widened and parallel to before narrower apex, setose in distally. Tegumen uniform medially; lateral arm dilated, with a broad anterior emargination. Costal part of valva with basal 1/3 uniform in width except wider base, distal 2/3 dilated, with fine hairs distally, with several denticles apically, forming a spine at ventral corner; costal band banded; transtilla bilobed: dorsal lobe subtriangular, ventral lobe longer, banded. Sacculus elongate, triangular in basal 2/3, produced to a clavate process in distal 1/3, with a setose process at base; dorsal margin elliptically folded ventrad; ventral margin elliptically folded dorsad. Saccus elongate, ~3× length of uncus, wide at base, gradually narrowed from base to rounded apex. Juxta weakly sclerotised, U-shaped, with lateral arms extended outward apically. Aedeagus > 2× length of costal part of valva, tubular in basal 2/5, then produced to a slender bar in distal 3/5, dilated before apex; with fine wrinkles from before middle to before apex, which beginning with a thorn; cornuti being two or three spines, placed at before apex.

Female genitalia (Fig. 30). Papillae anales short and broad. Apophyses posteriores 3× length of apophyses anteriores. Eighth sternal plate with a quadrate median groove, laterally with a triangular plate narrowed to posterolateral corner, lined with long setae on posterior margin. Lamella antevaginalis with ventral surface banded, arched inward on anterior margin, gently produced on posterior margin, with dorsal surface banded, arched inward on posterior margin, gently produced on anterior margin. Antrum subtrapezoidal, posterolaterally triangularly produced. Ductus bursae with posterior 1/2 sclerotised, anterior 1/2 membranous; appendix bursae arising from posterior 1/2 of ductus bursae, heavily sclerotised. Corpus bursae membranous, pyriform; signum small, spinelike, with a subtriangular base.

##### Etymology.

The specific epithet is derived from the Latin *productus*, referring to the elongate sacculus.

##### Distribution.

China (Sichuan Province).

#### Meleonoma quadrata sp. nov.

Taxon classificationAnimaliaLepidopteraAutostichidae

10999DBC-8987-5B29-A0DA-B005ADD7FA49

https://zoobank.org/B98D15D6-09BD-49F8-A062-064D32D48171

Figs 9, 22

##### Type material.

***Holotype*: China** • ♂; Yunnan Prov., Xishuangbanna, Menglun; 750 m a.s.l.; 24 X.2010; Bingbing Hu et al. leg.; slide no. ZXJ19538, in NKU. ***Paratypes***: • 1 ♂; same data as for holotype, in NKU 3 ♂; Xishuangbanna, Lvshilin; 29 V–5 VI.2015; Zhenguo Zhang leg.; slide no. ZXJ19091, in NKU.

##### Diagnosis.

The new species is similar to *M. torophanes* (Meyrick, 1935) in male genitalia. It can be distinguished by the forewing with a median costal spot; the costa with a triangular process at base, and the apex of the sacculus with a quadrate process in the male genitalia. In *M. torophanes*, the forewing has a median fascia (Wang 2006b: 152, fig. 130); the costa lacks a process at base, and the sacculus has a digitate process at the apex (Wang 2006b: 152, fig. 256).

##### Description.

Forewing length 6.0 mm (Fig. 9).

***Head*** yellow, tipped with greyish brown on vertex and laterally on occiput. Labial palpus with first and second segments dark brown on outer surface, yellow on inner surface; second segment forming a dark brown ring at apex; third segment ~1/2 length of second segment, with sparse dark brown scales. Antenna with scape mixed with black scales; flagellum black on dorsal surface, yellow on ventral surface.

***Thorax*** black, yellow apically; tegula black basally, yellow distally. Forewing with costal margin arched, apex rounded; ground colour dark brown; with a yellow spot at base; median costal spot yellow, subrectangular; distal spot subtriangular, at distal 1/4, narrowed posteriorly; plical spot black, placed at distal 1/3 of fold, with a yellow spot on its outer side, reaching end of fold; discal and discocellular spots black, placed at basal 3/5 and at outer margin of cell, respectively, with yellow scales on outer side, the latter not clearly distinguished from ground colour, touching with discal spot; dorsum with a yellow spot near base; fringe greyish brown, with a yellow basal line. Hindwing and fringe brown.

***Legs*** yellow, with exceptions on ventral surface: coxa and femur of foreleg with scattered dark brown scales, femur of midleg dark brown apically, tibiae of fore- and midlegs dark brown except yellow at middle and at apex, tarsus of foreleg dark brown except yellow at base of basal tarsomere and yellow at apices of basal two tarsomeres and yellow at apical tarsomere, tarsus of midleg with basal four tarsomeres dark brown except yellow at apices, tibia of hindleg with scattered dark brown scales, tarsus with basal three tarsomeres dark brown except yellow at apices.

***Abdomen***: Male genitalia (Fig. 22). Uncus rectangular, setose. Tegumen narrowed medially; lateral arm widened anteriorly, blunt at apex. Costal part of valva narrow at base, gradually widened to rounded apex, with a row of stout setae extending horizontally from near base to basal 2/5, with dense fine hairs distally; ventral margin gently produced medially, ending with a spine; costal band reaching at basal 3/5 of costal part of valva, with a triangular process at base; transtilla weakly sclerotised, narrowed extended toward middle, rounded at apex. Sacculus transversely trapezoidal, wide at base, narrowed from base to before apex; apex with a quadrate process, heavily sclerotised, its apex concave medially; dorsal margin folded ventrad, forming a sclerite. Saccus ~2× as long as uncus, wide at base, narrowed from base to rounded apex. Juxta stout, U-shaped. Aedeagus ~ 1.5× length of costal part of valva, tubular in basal 2/5; distal 3/5 narrower, apex concave medially, forming two apical processes; a sclerotised plate extending from middle to before apex, its ventral surface composed of a row of spines, forming a thorn at apex.

Female unknown.

##### Etymology.

The specific epithet is derived from the Latin *quadratus*, referring to the sacculus with a quadrate process at the apex.

##### Distribution.

China (Yunnan Province).

#### Meleonoma renaria sp. nov.

Taxon classificationAnimaliaLepidopteraAutostichidae

FAD0620D-92DC-549B-92D5-765A6A52AEEA

https://zoobank.org/71F65D89-A8F0-4AAE-AC60-6FBAE177D1A4

Figs 10, 23, 31

##### Type material.

***Holotype*: China** • ♂; Yunnan Prov., Nujiang, Mt. Gaoligong, Qinlangdang; 27.69°N, 98.27°E; 380 m a.s.l.; 28 V.2017; Kaijian Teng et al. leg.; slide no. ZXJ19520, in NKU. ***Paratypes***: • 3 ♂; same data as for holotype; slide no. ZXJ19521, in NKU 1 ♂ 1 ♀; 30–31 V.2017; other data same as for holotype; slide no. ZXJ19533 ♀, in NKU.

##### Diagnosis.

The new species is similar to *M. furcellata* (Wang, 2004) in male genitalia. It can be distinguished in the male genitalia by the costal part of the valva with a carina running from base to before basal 1/3 above the ventral margin, the sacculus produced to a triangular process distally, and the eighth sternal plate semi-circularly convex on the anterior margin in the female genitalia. In *M. furcellata*, the carina of the costal part of the valva extends from base to near the apex above the ventral margin, the sacculus produces to a rounded process (Wang 2004: 226, fig. 6), and the eighth sternal plate is straight on the anterior margin (Wang 2004: 226, fig. 17).

##### Description.

Forewing length 5.5–6.0 mm (Fig. 10).

***Head*** yellow, black on vertex. Labial palpus yellow; second segment with a black ring at apex; third segment ~2/3 length of second segment, with a black line from base to apex on ventral surface, with black scales in distal 1/3 on dorsal surface. Antenna black on dorsal surface, yellow on ventral surface; scape yellow apically on dorsal surface; flagellum alternated with black on ventral surface.

***Thorax*** and tegula black. Forewing black, tinged with orange yellow scales; median costal spot orange yellow, situated before middle, subrectangular; distal spot orange yellow, triangular, at distal ~1/4, mixed with sparse black scales; plical spot black, at basal 3/5 of fold, with an orange yellow spot on its outer side; discal and discocellular spots black, placed at basal 3/5 and at outer margin of cell, respectively, the former with a small orange yellow spot on outer side; in one individual, median costal spot represented by a median fascia, diffused with black scales downwards to middle; plical, discal and discocellular spots black, not clearly distinguished from ground colour; fringe black. Hindwing and fringe dark brown.

***Legs*** yellow, with exceptions on ventral surface: femur of foreleg with scattered dark brown scales, tibia dark brown mixed with yellow scales, tarsus dark brown except yellow at base of basal tarsomere and at apices of basal two tarsomeres and yellow at apical tarsomere, tibia of midleg dark brown, tarsus black except yellow at apices of basal two and apical one tarsomeres, tibia of hindleg dark brown except whitish yellow at middle and at apex, tarsus with basal four tarsomeres black except whitish yellow at apices.

***Abdomen***: Male genitalia (Fig. 23). Uncus triangular. Tegumen narrowed medially; lateral arm uniform except narrower apex. Costal part of valva uniform in basal 1/3, widened from basal 1/3 to middle, thereafter narrowed to apex, with fine hairs distally; apex rounded, with three or four spines, different in number in left and right of costal parts of valvae; a carina arising from base to before basal 1/3 above ventral margin, reaching at 2/3 width of costal part of valva; costal band gently concave basally, produced distally; transtilla short, rounded at apex. Sacculus wide at base, gradually narrowed in basal 2/3, then sharply narrowed and produced to a triangular process distally; ventral margin with basal 2/3 folded dorsad. Saccus as long as uncus, narrowed from wide base to narrowly rounded apex. Juxta U-shaped. Aedeagus nearly as long as costal part of valva, tubular in basal 7/10, produced to a ventral bar distally, curved toward base before apex, with several sclerites apically; cornuti being a cluster of spines.

Female genitalia (Fig. 31). Papillae anales rectangular, setose on dorsal surface. Apophyses anteriores ~2/3 as long as apophyses posteriores. Eighth sternal plate spiculate; triangularly concave medially on posterior margin, semi-circularly convex on anterior margin. Lamella antevaginalis large, spiculate, surrounding ostium bursae, reniform laterally, roundly produced posterolaterally, narrowly banded anteriorly. Ductus bursae heavily sclerotised in posterior 1/2, membranous in anterior 1/2, widened anteriorly; appendix bursae arising from near entrance of corpus bursae, sclerotised at base. Corpus bursae ovate; signum absent.

##### Etymology.

The specific epithet is derived from the Latin *renarius*, referring to the lamella antevaginalis being reniform laterally.

##### Distribution.

China (Yunnan Province).

#### Meleonoma simililunformis sp. nov.

Taxon classificationAnimaliaLepidopteraAutostichidae

399DDACE-D0B0-5FFF-8235-CD7A4EDF8600

https://zoobank.org/390195D1-05F1-4EEA-88E2-C4F0A75F986E

Figs 11, 24

##### Type material.

**Holotype: China** • ♂; Guizhou Prov., Maolan, Banzhai Village; 25.23°N, 108.03°E; 530 m a.s.l.; 14 VIII.2018; Meiling Zheng et al. leg.; slide no. ZXJ19633, in NKU. ***Paratypes***: • 3 ♂; 8–13 VIII.2018; other data same as for holotype, in NKU.

##### Diagnosis.

The new species is similar to *M. luniformis* (Wang, 2006a) in both forewing pattern and male genitalia. It can be distinguished by the apex of the costal part of the valva slightly concave below the dorsal corner, and the process of the sacculus not produced dorsally. In *M. luniformis*, the apex of the costal part of the valva is straight below the dorsal corner, and the process of the sacculus is roundly produced dorsally (Wang 2006a: 12, fig. 4).

##### Description.

Forewing length 4.0–4.5 mm (Fig. 11).

***Head*** dark brown, whitish yellow on frons. Labial palpus yellow; second segment with scattered dark grey scales, denser in distal 1/2, forming a dark grey ring at apex; third segment ~2/3 length of second segment, with scattered dark brown scales, denser from middle to before apex on dorsal surface. Antenna dark brown on dorsal surface, yellow on ventral surface; scape yellow apically on dorsal surface; flagellum alternated with yellow on ventral surface.

***Thorax*** and tegula dark brown. Forewing dark brown, with costal margin arched, apex narrowly rounded; median fascia yellow, from before middle of costal margin gradually widened and obliquely outward to before tornus; distal spot large, at distal ~1/3, yellow, mixed with dark brown scales, posteriorly diffused to median fascia, forming a large crescent patch on forewing; fringe greyish black, with a whitish yellow basal line. Hindwing and fringe dark grey.

***Legs*** whitish yellow, with exceptions on ventral surface: coxa of foreleg with sparse dark brown scales, femur dark brown, mixed with whitish yellow scales distally, femora of mid- and hindlegs with sparse dark brown scales, tarsus of foreleg dark brown except whitish yellow at base of basal tarsomere and at apices of basal two and apical one tarsomeres, tarsus of midleg dark brown except yellow at apices of basal two and apical one tarsomeres, tarsus of hindleg with basal four tarsomeres dark brown except yellow at apices, all tibiae dark brown except yellow at middle and at apex.

***Abdomen***: Male genitalia (Fig. 24). Uncus lanceolate, with long setae, apex rounded. Tegumen with lateral arms narrowly banded. Costal part of valva dilated in foliaceous shape, with a small process at base near ventral margin, with fine hairs distally; apex with two or three small teeth, slightly concave inward below dorsal corner; costa humped near middle; transtilla short, narrowed toward middle, rounded at apex. Sacculus widest at base, narrowed to ~2/3, distal 1/3 being a clavate process, uniform in width except slighter narrower apex, pointed at apex. Saccus wide at base, gradually narrowed to rounded apex, shorter than uncus. Juxta U-shaped, lateral lobe narrowed distally. Aedeagus longer than costal part of valva, tubular in basal 2/3, widest at basal 1/3, with a small rounded process dorsally at basal 2/3, distal 1/3 produced to a narrow band, dilated and pointed at apex; cornuti being two spines in vesica, different in size.

Female unknown.

##### Etymology.

The specific epithet is derived from the Latin *simil*- and the specific epithet luniformis, indicating the similarity of the two species.

##### Distribution.

China (Guizhou Province).

#### Meleonoma strena sp. nov.

Taxon classificationAnimaliaLepidopteraAutostichidae

F4EB864B-60F0-5BB0-989E-B5D087B7B11A

https://zoobank.org/1FDE51F5-AB9E-4D58-A321-A99C2C8C693B

Figs 12, 25, 32

##### Type material.

***Holotype*: China** • ♂; Yunnan Prov., Nujiang, Gongshan County, Pukawang Village; 27.84°N, 98.32°E; 1335 m a.s.l.; 7 VI.2017; Kaijian Teng et al. leg.; slide no. ZXJ19526, in NKU. ***Paratypes***: • 5 ♂; same data as for holotype, in NKU 8 ♂ 1 ♀; 5–11 VI.2017; other data same as for holotype; slide no. ZXJ19534, in NKU.

##### Diagnosis.

The new species is similar to *M. producta* sp. nov. in male genitalia by the sacculus produced to a clavate process distally. It can be distinguished by the costal part of the valva without denticles apically, the saccus 2× the length of the uncus, and the aedeagus produced to a slender bar in distal 4/5. In *M. producta*, the costal part of the valva bears several denticles apically, the saccus is 3× the length of the uncus, and the aedeagus produces to a slender bar in distal 3/5.

##### Description.

Forewing length 6.0–8.0 mm (Fig. 12).

***Head*** pale yellow, tipped with black on occiput. Labial palpus pale yellow; first segment dark brown on outer surface; second segment with scattered dark brown scales, forming a ring at apex; third segment ~ 2/3 length of second segment. Antenna dark brown on dorsal surface, pale yellow on ventral surface; flagellum alternated with pale yellow on ventral surface.

***Thorax*** and tegula dark brown. Forewing dark brown, with costal margin arched, apex narrowly rounded; median fascia whitish yellow, from middle of costal margin extending obliquely outward to before tornus, widened posteriorly; distal spot whitish yellow, inverted triangular, at distal ~1/3, with a small dark brown dot in middle anteriorly; plical spot black, at basal 3/5 of fold, placed at inner margin of median fascia; discal and discocellular spots black, placed at inner and outer margins of median fascia, respectively; fringe whitish yellow. Hindwing brown, fringe whitish yellow.

***Legs*** whitish yellow, with exceptions on ventral surface: foreleg dark brown except coxa mixed with whitish yellow scales, tibia whitish yellow apically, tarsus whitish yellow at base of basal tarsomere and at apices of basal two and whitish yellow at apical tarsomere, femora of mid- and hindlegs with sparse dark brown scales, tibia of midleg dark brown except whitish yellow at middle and at apex, tibia of hindleg with sparse greyish brown scales, tarsus of midleg with basal four tarsomeres dark brown except whitish yellow at apices, tarsus of hindleg with sparse greyish brown scales on basal two tarsomeres.

***Abdomen***: Male genitalia (Fig. 25). Uncus subrectangular, with dense long setae in distal 1/2. Tegumen narrowed medially; lateral arm uniform, blunt at apex. Costal part of valva narrow at base, gradually widened to apex, with stout setae before middle, with fine hairs distally, with a spine at ventral angle; ventral margin heavily sclerotised, gently produced medially, with a carina from base to basal 1/2 above ventral margin; costal band with a small incision at base, concave medially, produced distally, reaching before tip of costal part of valva; transtilla narrowed toward middle, pointed at apex. Sacculus wide at base, narrowed to basal 3/5, distal 2/5 produced to a clavate process, rounded at apex; dorsal margin folded ventrad, forming a band. Saccus broad, 2× length of uncus. Juxta weakly sclerotised, U-shaped. Aedeagus slightly longer than costal part of valva, tubular in basal 1/5, distal 4/5 produced to a slender bar; cornuti stout, being two spines, connected to each other and with fine wrinkles medially.

Female genitalia (Fig. 32). Papillae anales subquadrate, with sparse setae dorsally. Apophyses posteriores ~ 2.5× length of apophyses anteriores. Eighth sternal plate with a narrow median groove, laterally with a subovate plate narrowed to posterolateral corner, lined with long setae on posterior margin. Antrum rectangular, broadly concave medially on posterior margin, posterolaterally produced to a subovate process extending forward. Ductus bursae membranous, widened in anterior 2/3; ductus seminalis arising from junction of ductus bursae and corpus bursae. Corpus bursae small, shorter than ductus bursae; signum rhombus-shaped, with a sclerotised carina mesially.

##### Etymology.

The specific epithet is derived from the Latin *strenus*, referring to the stout cornuti.

##### Distribution.

China (Yunnan Province).

#### Meleonoma utricularis sp. nov.

Taxon classificationAnimaliaLepidopteraAutostichidae

35AF054B-9B0C-57A2-9068-FF3ED73F7D8C

https://zoobank.org/08632643-65E2-42A4-9476-F060CA06E768

Figs 13, 26, 33

##### Type material.

***Holotype*: China** • ♂; Xizang Autonomous Region [Tibet], Motuo County [Mêdog], Motuo Road; 29.66°N, 95.49°E; 2076 m a.s.l.; 7 VIII.2019; Mujie Qi leg.; slide no. ZXJ19490, in NKU. ***Paratypes***: • 3 ♀; same data as for holotype; slide no. ZXJ19530, in NKU 1 ♀; 6 VIII.2019; other data same as for holotype; slide no. ZXJ19509, in NKU 1 ♂; • Xizang Autonomous Region [Tibet], Motuo County [Mêdog]; 1016 m a.s.l.; 5 VIII.2018; Mujie Qi leg.; slide no. ZXJ19492, in NKU.

##### Diagnosis.

The new species is similar to *M. longisacca* sp. nov. in both forewing pattern and male genitalia. It can be distinguished in the male genitalia by the costal part of the valva distinctly concave basally on the ventral margin, the saccus slightly longer than the uncus, and the cornutus absent. In *M. longisacca*, the costal part of the valva is straight basally on the ventral margin, the saccus is ~ 2.5× length of the uncus, and the cornuti are present.

##### Description.

Forewing length 5.5–7.0 mm (Fig. 13).

***Head*** dark brown, yellow laterally on occiput. Labial palpus with first and second segments dark brown on outer surface, yellow on inner surface, with a dark brown ring at apex on second segment; third segment yellow, with sparse dark brown scales. Antenna dark brown, scape yellow apically.

***Thorax*** and tegula dark brown. Forewing dark brown, mixed with yellowish white scales; median costal spot yellowish white, situated before middle; distal spot yellowish white, at distal ~1/3; plical spot black, with a yellowish brown spot on its outer side; discal and discocellular spots black, placed at basal 2/3 and at outer margin of cell, respectively, the former with a yellowish brown spot on its outer side; fringe dark brown, with a yellowish white basal line. Hindwing and fringe brown.

***Legs*** yellowish white, with exceptions on ventral surface: coxa of foreleg with sparse dark grey scales, femur and tibia dark grey, tarsus dark grey except yellowish white at base of basal tarsomere and at apices of basal two tarsomeres, femur of midleg dark grey apically, tibia dark grey except yellowish white at middle and at apex, tarsus dark grey except yellow at apices of basal three tarsomeres, hindleg with scattered dark grey scales.

***Abdomen***: Male genitalia (Fig. 26). Uncus uniform in basal 1/4, shrunken beyond basal 1/4, uniformly narrow to before basal 2/3, widened at basal 2/3, thereafter narrowed to narrowly rounded apex, setose. Tegumen uniform, banded. Costal part of valva narrow at base, widened to 1/2, dilated subquadrately in distal 1/2, with fine hairs distally; costal band reaching distal 1/3 of costal part of valva, with large setae basally; ventral margin heavily sclerotised, distinctly concave basally, produced distally, ending with a spine; transtilla clavate, rounded at apex. Sacculus triangular, wide at base, gradually narrowed to basal 2/3, distal 1/3 spine-shaped, stout, pointed at apex; dorsal margin heavily sclerotised in distal 3/5; ventral margin concave beyond middle, heavily sclerotised in distal 1/3. Saccus slightly longer than uncus, narrowed from wide base to narrowly rounded apex. Juxta weakly sclerotised, U-shaped. Aedeagus slightly longer than costal part of valva, bent medially; cornutus absent.

Female genitalia (Fig. 33). Papillae anales subquadrate, with sparse setae on dorsal surface. Apophyses anteriores ~1/3 length of apophyses posteriores. Eighth sternal plate concave medially and lined with long setae on posterior margin. Lamella antevaginalis with ventral surface banded anteriorly, arched inward laterally; dorsal surface subrectangular, with tiny teeth on posterior margin. Antrum narrowed posteriorly. Ductus bursae membranous, evenly wide; appendix bursae arising from anterior part of ductus bursae. Corpus bursae small, not distinctly separated from ductus bursae; signum absent.

##### Etymology.

The specific epithet is derived from the Latin utricularis, referring to the small corpus bursae.

##### Distribution.

China (Xizang Autonomous Region [Tibet]).

## Supplementary Material

XML Treatment for Meleonoma anisomorpha

XML Treatment for Meleonoma apicitriangula

XML Treatment for Meleonoma bisecta

XML Treatment for Meleonoma corniculata

XML Treatment for Meleonoma longisacca

XML Treatment for Meleonoma ovaliuncatus

XML Treatment for Meleonoma plicativa

XML Treatment for Meleonoma producta

XML Treatment for Meleonoma quadrata

XML Treatment for Meleonoma renaria

XML Treatment for Meleonoma simililunformis

XML Treatment for Meleonoma strena

XML Treatment for Meleonoma utricularis

## Additional information

### Use of AI

No use of AI was reported.

### Author ORCIDs

Xiaoju Zhu https://orcid.org/0000-0002-4590-8480

Shuxia Wang https://orcid.org/0000-0002-9316-6661

### Data availability

All of the data that support the findings of this study are available in the main text.

## References

[B1] Li HH (2002) The Gelechiidae of China (Lepidoptera, Gelechioidea) (I). Nankai University Press, Tianjin, 538 pp. [In Chinese]

[B2] Lvovsky AL (2010) A new genus of broad-winged moths of the family Cryptolechiidae (*Acryptolechia*, Lepidoptera, Gelechioidea) from Southeastern Asia. Entomological Review 90(2): 255–258. 10.1134/S0013873810020119

[B3] Meyrick E (1910) Notes and descriptions of Indian Microlepidoptera. Records of the Indian Museum 5: 217–232. 10.5962/bhl.part.10499

[B4] Meyrick E (1914) Exotic Microlepidoptera 1: 225–288. 10.5962/bhl.title.9241

[B5] Meyrick E (1921) Exotic Microlepidoptera 2: 385−416. 10.5962/bhl.title.9241

[B6] Meyrick E (1935) List of Microlepidoptera of Chekiang, Kiangsu and Hunan. In: Caradja A, Meyrick E (Eds) Materi alien zu einer Microlepidopteran Fauna der chinesischen Provinzen Kiangsu, Chekiang und Hunan. Friedlander & Sohn, Berlin, 44–96.

[B7] Wang SX (2003) A study of *Cryptolechia* Zeller (Lepidoptera: Oecophoridae) in China (I), with descriptions of fifteen new species. Entomologia Sinica 10(3): 195–213. 10.1111/j.1744-7917.2003.tb00384.x

[B8] Wang SX (2004) A study of *Cryptolechia* Zeller (Lepidoptera: Oecophoridae) in China (II): Species from Guizhou Province. Entomologia Sinica 11(3): 219–233. 10.1111/j.1744-7917.2004.tb00242.x

[B9] Wang SX (2006a) The *Cryptolechia* Zeller (Lepidoptera: Oecophoridae) of China (III): Checklist and descriptions of new species. Zootaxa 1195(1): 1–29. 10.11646/zootaxa.1195.1.1

[B10] Wang SX (2006b) Oecophoridae of China (Insecta: Lepidoptera). Science Press, Beijing, 258 pp.

[B11] Wang QY, Li HH (2020) Phylogeny of the superfamily Gelechioidea (Lepidoptera: Obtectomera), with an exploratory application on geometric morphometrics. Zoologica Scripta 49(3): 307–328. 10.1111/zsc.12407

[B12] Wang SX, Zhu XJ, Zhao BX, Yang XF (2020) Taxonomic review of the genus *Meleonoma* Meyrick (Lepidoptera: Autostichidae), with a checklist of all the described species. Zootaxa 4763(3): 371–393. 10.11646/zootaxa.4763.3.3 33056854

[B13] Wang SX, Zhu XJ, Tao ZL (2021) Study of the genus *Meleonoma* Meyrick, 1914 (Lepidoptera: Autostichidae) from China (III), with descriptions of eighteen new species. Zootaxa 4995(2): 303–333. 10.11646/zootaxa.4995.2.5 34810569

[B14] Zhu XJ, Wang SX (2022) Taxonomy of the genus *Meleonoma* Meyrick, 1914 (Lepidoptera: Autostichidae) from China (IV), with descriptions of twelve new species. Zootaxa 5087(4): 501–521. 10.11646/zootaxa.5087.4.1 35391273

